# Killer whale innovation: teaching animals to use their creativity upon request

**DOI:** 10.1007/s10071-022-01635-3

**Published:** 2022-09-20

**Authors:** Heather Manitzas Hill, Myriam Weiss, Isabelle Brasseur, Alexander Manibusan, Irene R. Sandoval, Todd Robeck, Julie Sigman, Kristen Werner, Kathleen M. Dudzinski

**Affiliations:** 1 St. Mary’s University, San Antonio, Texas USA; 2 Marineland, Antibes, France; 3 SeaWorld Florida, Orlando, FL USA; 4 SeaWorld Texas, San Antonio, TX USA; 5 Dolphin Communication Project, Port Saint Lucie, Florida USA

**Keywords:** Killer whale (*Orcinus orca*), Creativity, Individual differences, Flexible thinking, Torrance Tests

## Abstract

Thinking flexibly is a skill that enables animals to adapt to changing environments, which enhances survival. Killer whales, *Orcinus orca*, as the ocean apex predator display a number of complex cognitive abilities, especially flexible thinking or creativity when it comes to foraging. In human care, smaller dolphins and other marine mammals have been trained to think creatively while under stimulus control. The results of these previous studies have demonstrated that bottlenose dolphins, *Tursiops truncatus*, can create original behaviors in response to an innovative cue. We trained and tested a total of nine killer whales from two different facilities on the innovate concept, using the same methodology. The killer whales ranged in age from 5 to 29 yrs with 4 females and 5 males. The results indicate that the killer whales demonstrated high fluency, originality, some elaboration, and flexibility in their behaviors. Individual variability was observed with younger animals demonstrating more variable behaviors as compared to the older animals. Males seemed to display less complex and lower energy behaviors as compared to females, but this impression may be driven by the age or size of the animal. These results support existing evidence that killer whales are dynamic in their thinking and behavior.

**Supplementary Information:**

The online version contains supplementary material available at 10.1007/s10071-022-01635-3.

## Introduction

Many species demonstrate unique or creative variations of common behaviors expressed for communication or as used in foraging, socialization, and play. For example, killer whales (*Orcinus orca*) in managed care have been observed using fish pieces to lure seagulls close enough to catch (reviewed by Paulos et al. 2010). Similarly, bottlenose dolphins (*Tursiops* sp.) engage in locomotor play such as surfing, where they ride pressure waves produced by boats, ships, or other cetaceans (e.g., Paulos et al. 2010). Bottlenose dolphins in managed care often play with human-provided objects (e.g., balls, buoys, mats) and self-made bubbles (e.g., clouds, rings, trails), which is similar to play behaviors engaged in by wild dolphins with feathers, seaweed, sponges, and bubbles (KMD, personal observation, 1997–2002; Bateson 2014; Greene et al. 2011; Kuczaj and Eskelinen 2014a; Kuczaj and Highfill 2005). Other novel delphinid behaviors include sponge and bubble use during foraging by bottlenose dolphins (Smolker et al. 1997; Fertl and Wilson 1997), ice fishing techniques used to capture seals and penguins by killer whales (Visser et al. 2007), and intentional beach stranding to catch prey by killer whales in the Crozet Archipelago and bottlenose dolphins in Western Australia (Guinet 2011; Guinet & Bouvier 1995; Sargeant et al. 2005) and in South Carolina, USA (Duffy-Echevarria et al. 2008; Rigley et al. 1981).

Behavioral flexibility is often seen in foraging (Guinet and Bouvier 1995; Ramos et al. 2021; Rigley et al. 1981; Sargeant et al. 2005; Smolker et al. 1997; Visser et al. 2007) and play contexts (Hill and Ramirez 2014; Hill et al. 2017; Kuczaj et al. 2006) for aquatic mammals, as well as for terrestrial (Burghardt 2005, 2011) and avian species (O’Hara and Auersperg 2017; Ortega and Bekoff 1987). Flexibility in behavior suggests the presence of adaptive functioning in non-human animals (Bateson 2014; Herman 2006; Pryor and Chase 2014). Flexible thinking is considered indicative of complex cognitive functioning as exhibited by non-human animals, which also is described as being creative (Kaufman 2021; Kaufman and Kaufman 2004; Kuczaj and Eskelinen 2014a; Pryor and Chase 2014). In humans, creativity can manifest in a variety of ways, through art, expression, thought, and words (Gardner 1993; Guilford 1966; Kaufman and Baer 2004).

Although creativity in non-human animals may be difficult to define (Plucker and Makel 2010), it is a construct that may explain the gap between antecedent events and resulting observable responses. An antecedent event may elicit a creative act that might represent a novel and functional behavior never before emitted. The non-human animal’s ability to produce a creative behavior is dependent on its knowledge and cognitive abilities; animals can only produce behaviors that stem from what they have already learned or are capable of learning (Bailey et al. 2007; Kubina et al. 2006). Similarly, executing a new idea accesses different cognitive processing as compared to developing the new idea, as discussed for non-human animals (Bateson 2014) and for humans (Kubina et al. 2006).

Among the tests available to examine creativity in animals, the Torrance Tests of Creative Thinking (TTCT) is the most widely applied (see Torrance 1974; Kaufman 2021; Kaufman and Kaufman 2004; Kaufman and Baer 2006). TTCT was developed from Guilford’s approach to the theory of creativity (Guilford 1966). Both researchers defined four categorical variables to assess creativity: fluency, flexibility, originality, and elaboration. Fluency is the number of ideas generated. Flexibility represents the ability to produce many different types of ideas, as well as how many categories into which those ideas fit. Originality is being able to produce unique ideas. Elaboration is the ability to expand on those ideas with detail and examples.

The TTCT were not originally created for use with non-human animals; however, Kaufman and Kaufman (2004) suggested that it might be a reasonable foundation from which to assess creativity in non-human animals. The idea that non-human animals can vary their behavior when asked to do so by humans was first investigated by Pryor et al. (1969; Pryor and Chase 2014), who trained rough-toothed dolphins (*Steno bredanensis*) to respond to a discriminative stimulus, or S_D_, with a novel behavior. Their goal was to increase the behavioral repertoire of the dolphins; Pryor et al. (1969) used the S_D_ to request a novel action, one not before performed. Although Pryor’s goal was to add actions to the animals’ behavioral repertoire, Pryor and colleagues succeeded in developing a training method that produced behaviors that could be considered creative. Their method was originally used as a game to train dolphins (“innovate”) but eventually turned into a training technique used for a variety of animals, including bottlenose dolphins, dogs (*Canis lupus familiaris*), and California sea lions (*Zalophus californianus*) as summarized by Dudzinski and colleagues (2018). Kuczaj and Eskelinen (2014b) expanded upon Pryor’s original study, using an S_D_ to request creative responses (i.e., do something that you have not done before) from bottlenose dolphins. Each session had a trainer presenting the “create” S_D_ to a dolphin followed by the dolphin’s response; the process was repeated until the dolphin failed to produce a new behavior or the trainer ended the session (Kuczaj and Eskelinen 2014b).

Following recommendations in Kaufman and Kaufman (2004) to apply the constructs of creativity as measured by the TTCT to animal creativity, Kuczaj and Eskelinen (2014b) assessed three of the constructs indirectly: fluency, flexibility, and originality. The results of this initial study demonstrated that the male bottlenose dolphins produced a number of different behaviors (fluency) from different categories of behaviors, such as the type and energy level exhibited (flexibility). Moreover, each dolphin produced novel behaviors or novel sequences of behaviors (originality). Kuczaj and Eskelinen (2014b) did not refer to their measures as these constructs; however, the operational definitions utilized align nicely with constructs of creativity established by Guilford (1966) and expanded upon by Torrance (1974) and Kaufman and Kaufman (2004). Kaufman and Kaufman (2004) did note that the last construct, elaboration, would be difficult to assess in animals. Typically, elaboration is assessed by the degree of detail or embellishment that is provided when completing a standardized test stimulus. In the case of the “innovate” behavior, the animal must produce a behavior different from what had been produced previously or if conservative, a truly novel, never-before trained or performed behavior. Once the concept is learned, elaboration may require additional training, which would potentially change the nature of the acceptable criteria that are reinforceable (Kaufman & Kaufman 2004). When Guilford (1966) and Torrance (1974) developed their theory and assessments, respectively, their frame of reference was to evaluate intelligence and creativity in humans under spontaneous contexts (i.e., no training needed). In humans, the creative output can be increased through reinforcement but will extinguish in specific contexts if reinforcement is withheld and may not always transfer to other contexts (reviewed by Eisenberger et al. 1998; Eisenberger and Cameron 1996). The idea of training “creativity” with reinforcements and placing it under stimulus control creates a different context in which creativity is expressed and potentially interpreted.

Another consideration with regard to creativity in animals is the influence of social relationships and societal structure on the expressed actions and behaviors of a species. As a matrilineal society, killer whale social structure is shaped by maternal kinship and strong natal philopatry with hierarchically structured units (Baird 2000; Esteban et al. 2015). Despite the consistent matrilineal characteristic of the species, individual differences appear to exist among kin-based pods; Nousek et al. (2006) found vocal variations between free-ranging individual northern resident killer whales that were constantly associated with each other, who also engaged in group-specific vocalizations. Thus, not only are there individual differences in killer whale vocalizations, but also individuals can potentially distinguish between highly similar shared calls of their matrilineal relatives (Nousek et al. 2006). Dahlheim and Awbrey (1982) had found similar results identified in distinct acoustic groups of individual killer whales in managed care at multiple facilities. In addition to individually distinct vocalizations, killer whales globally present distinct hunting strategies (Kuczaj et al. 1998; Visser et al. 2007; Guinet 2011; Guinet and Bouvier 1995), which supports the notion that individual variation and flexibility in foraging strategies are prevalent (Saulitis et al. 2000; Similä and Ugarte 1993) and may reflect creative differences.

The purpose of this study was to investigate the possibility of killer whale creativity based on the four criteria proposed by Guilford (1966) and operationalized by Torrance (1974): fluency, flexibility, originality, and elaboration. In collaboration with trainers from two facilities, killer whales were taught an S_D_ for creating – to offer a behavior not presented in the session (the action could be from the animals’ repertoires or untrained). All sessions were video recorded to facilitate behavior coding and reliability. The following overarching research questions were examined:

1. Do we have evidence of creativity in killer whales under stimulus control, as measured based on the four constructs operationalized by Torrance (1974) and extended with the current study, as proposed by Kaufman and Kaufman (2004) and initially investigated by Kuczaj and Eskelinen (2014b)?
2. Assuming the answer to our first question is yes, is there evidence for variation in killer whale creativity based on age or sex?

## Method

### Study subjects

Nine killer whales were included in this study from SeaWorld Texas (SWT) and Marineland France (MLF) (Table 1). At SWT, TAK was the matriarch; at MLF, WIK was the matriarch. KYU, TUA, and INO were mature males while SAK, KAM were immature females and MOA and KEI were immature males (Table 1).

**Table 1:** Demographic and test session details for the study subjects

Animal ID	Age (Y) at testing	Sex	Facility	# Test sessions total*	Relationship
TAK	28	Female	SWT	3	Matriarch
KYU	27	Male	SWT	3	
TUA	19	Male	SWT	3	
SAK	9	Female	SWT	3	Offspring of TAK
KAM	5	Female	SWT	3	Offspring of TAK
WIK	19	Female	MLF	6	Matriarch
INO	21	Male	MLF	6	
MOA	9	Male	MLF	6	Offspring of WIK
KEI	6	Male	MLF	8	Offspring of WIK

*MLF* is Marineland France. *SWT* is SeaWorld Texas. *Y* is years. Age is listed for each killer whale at the time of study testing

*For this study, we only used the first three sessions to facilitate inter-animal comparisons

### Training and testing protocols

The same training protocol was utilized at each facility with training at SWT (September 2017–2018) occurring about two years before the training at MLF (July 2019–2020). Each killer whale was trained to respond to the innovate behavioral S_D_, or hand cue, presented by a trainer. At SWT, killer whales were trained by a team with a lead trainer designated for each killer whale. At MLF, the majority of training for this innovate behavioral S_D_ was conducted by a primary trainer per animal. (See Dudzinski et al. (2018) for a detailed presentation of the protocols and limitations of training the innovate cue.) Training sessions allowed the killer whales to become familiar with this cue. Each killer whale learned the innovate S_D_, which was to respond with different behavior than previously performed at each previous innovate S_D_. Training lasted about a year for both facilities with testing occurring after training was completed. Training consisted of multiple sessions over time (both within days and weeks) for each individual animal. A number of training sessions were recorded for all animals, but specific details regarding behavioral performance and reinforcements were collected only for five of the nine animals. These data will be assessed in a separate paper. During a training session, animals received multiple cues with early training including a fixed ratio schedule of continuous reinforcement. Trainers would prompt animals if they were repetitive in behavior responses, especially in early training. Any behavior was acceptable, as long as it was different from the immediately performed action. Once animals were responding consistently to the cue, several cues were given in succession before reinforcement was received, which consisted of food and secondary reinforcers (e.g., ice, rubdowns, etc.). Sessions were typically short (~ 5–8 min.) that was consistent with regular training. Trainers monitored animal performance to minimize frustration during the learning process (for more detail, see Dudzinski et al. 2018).

Testing was conducted once the killer whales were able to complete at least 8–10 trials during a training session with minimal errors (i.e., repeated a previously performed behavior in the session); a trial is the presentation of a single innovate S_D_. All animals produced different numbers of trials in a session, which meant a session did not have a standard number of trials across animals. During training, if an animal made more than three errors in a row then the trainers prompted the animal (i.e., hinted) or moved to a different behavior to avoid animal frustration. During testing, if an animal made more than three errors in a row (i.e., three independent S_D_ cues were given and the same behavior was presented consecutively three times after the first presentation of that behavior) then the session ended; no hinting was provided. All testing sessions were videotaped (at SWT: Sony Handycam HDR-CX405; at MLF: Kodak PIXPRO WPZ2) for later coding. Test sessions occurred primarily in the mornings and were completed for one killer whale at a time (i.e., not in tandem). At both SWT and MLF, a test session continued with the S_D_ given until one of three situations occurred: (1) the test animal repeated the same behavior three times in a row; (2) fish (primary reinforcement) was running low; or (3) the trainer terminated the session due to low motivation or for an excellent performance (i.e., no repeated behaviors or very different behaviors performed for each trial). Trainer discretion dictated session ends for the last two situations. When a different behavior was performed in response to the innovative S_D_, the killer whale received a randomly selected form of reinforcement from the trainer (i.e., fish, gelatin, rub down, water, ice).

At SWT, test sessions were conducted in October 2019 with each killer whale participating in three sessions. These SWT test sessions used a standard reinforcement procedure in which reinforcement was varied on both schedule (i.e., not every behavior was rewarded with a primary or secondary reinforcer after it was marked with a whistle bridge) and type (i.e., handful of fish, 1–2 herring, handful of ice, gelatin) regardless of the behavior performed; however, no increased magnitude of reinforcements was given during testing to control for response to quantity). At MLF, test sessions were conducted in the summer 2020, with each killer whale participating in 12 sessions. Six sessions used a standard reinforcement procedure with a consistent amount of reinforcement given regardless of behavior performed. Six additional sessions per killer whale were conducted using a variable reinforcement contingency that elicited greater motivation for novel or unusual behaviors. For both facilities, innovative cue requests were given immediately after a primary (e.g., fish) or secondary (e.g., ice, tactile) reinforcement was provided to the animal or the reinforcement was given after the animal responded to several innovate cues (i.e., intervals between cues ranged between immediate and length of time required by the animal to receive reinforcement). For this study, to facilitate direct comparison among all nine killer whales with respect to creativity variables (see “Variable definitions”), only the first three test sessions for each killer whale were included in these analyses. The first three test sessions for MLF animals were selected because the animals at SWT completed only three test sessions. This reduction in the inclusion of sessions facilitated direct comparison across all animals.

### Coding

Video recordings of all test sessions conducted at SWT were coded by two naïve scorers: AM and CLR (see “Acknowledgements”). One scorer, AM, coded all trials per session for each killer whale. The second scorer, CLR, coded 25% of the trials per session for each killer whale. Reliability for behaviors identified as the reinforced behavior was 100% in agreement. For the MLF test session videos, a trainer from MLF, MW, reviewed all test session videos to document all killer whale behavioral responses (i.e., behaviors accepted by trainers in response to the innovate S_D_.) and to translate audio narrative from French to English. The MLF trainer (MW) identified each behavior presented by each killer whale and identified whether the action was a repeated behavior, new to that session, or new to the animal’s repertoire. A second scorer, IS, coded 25% of the trials per session for each killer whale, resulting in 100% agreement.

The resulting correct behaviors were then coded for the four creativity variables assessed: fluency, flexibility, originality, and elaboration (see “Variable definitions” for categories within each variable and Table 2.). Two scorers coded each variable; AM coded the SWT data and IS coded the MLF data. For reliability, 25% of trials from each session per animal were re-coded by a trained research assistant, CLR, for correct behavioral data. The reliability using an inter-observer agreement calculation (inter-rater percent agreement) averaged 93.5% in agreement.

**Table 2:** Creativity variables defined

Creativity variable	Variable definition	Operational definitions	How measured in this study
Fluency	The number of different behaviors produced for correct trials per animal	Total number of behaviors performed (all trials in a session)	N_T_ (*total number of behaviors performed*)
		Number of reinforced (correct) behaviors from the total number of behaviors performed	N_R_ = “*correct/reinforced N*” So, proportion is N_R_/N_T_
		Total number of reinforced behaviors	N_R_
		Number of consecutively reinforced behaviors (trials) before a repeat	Consecutive new behaviors before a repeat (N_CNB_)
Flexibility	How many different types of behaviors presented by the animal(s)	Energy	High, moderate, low
		Type	Motor, Vocal, Bubbles, Single or Multiple
		Repertoire	Within existing behavioral repertoire or not (i.e., new)
Originality	The degree of uniqueness of a behavior exhibited by an animal. It is defined by how different the behavior is from the typical behavioral repertoire of the animal, the social population, or the species	This variable can be evaluated by examining the performed behavior in comparison to the performed behaviors of the individual, social group, or other animals studied on this task. More common behaviors are less original; less common behaviors are more original	(1) Behavior was produced (and reinforced) only once across **all sessions per animal** (behaviors were identified as single actions or complex/compound actions)
			(2) Behavior was produced only once across **all animals and all sessions**
			(3) Behavior was in the animal’s repertoire or not
Elaboration	The degree of complexity of the action performed	Simple (single) behaviors versus compound actions	(1) Single actions (2) Behavior sequences (3) Simultaneous actions

### Variable definitions

Creativity was measured following the application of the Torrance Tests of Creative Thinking (TTCT, Torrance 1974) and the four variables presented: fluency, flexibility, originality, and elaboration (Table 2). Several operational definitions were identified for each of the four variables and were either adapted from previous work with bottlenose dolphins (Kuczaj and Eskelinen 2014b) or developed from the human literature (Kaufman 2021; Kaufman and Kaufman 2004; Torrance 1974) (Table 2).

Fluency is the number of correct different behaviors presented within a session and is generally measured as the ratio of correct responses to the total number of innovate cues requested (Table 2). Four different operational definitions were measured to estimate fluency for each killer whale: (1) total number of reinforced (correct) behaviors, (2) number of reinforced (correct) behaviors from the total number of behaviors performed (percent correct), (3) total behaviors performed, and (4) highest number of consecutively reinforced behaviors before a behavior was repeated and not reinforced (Table 2).

Flexibility represents how many types of behaviors are exhibited in response to the innovate S_D_. Other researchers have coded behaviors into various categories including low versus high energy (Kuczaj and Eskelinen 2014b), and demonstration, husbandry, or natural behaviors (Yeater et al. 2014). We examined flexibility using three different categories: energy, type, and repertoire (Table 2). Energy was operationally defined by 10 different levels, beginning with low, moderate, and high, followed by different combinations of compound actions (Table 3). Low energy behaviors used low effort or low intensity (e.g., quiet whistle as interpreted by the trainer). Moderate energy behaviors took moderate effort, like a pectoral fin slap, or were performed with moderate intensity (e.g., mid-volume vocalizations as interpreted by the trainer). High energy behaviors generally took effort, like aerials, or were performed with high intensity (e.g., loudness for vocalizations as interpreted by the trainer). Flexibility’s type category was represented by behaviors identified as locomotor, vocal, bubbles or multiple (Table 2) and used a similar division of 10 different levels, such that different types of compound behaviors could be identified (e.g., vocal and motor, Table 3). The third categorical classification for flexibility was repertoire: was the behavior in the animal’s repertoire or not (Table 2).

**Table 3:** Fluency, flexibility, originality, and elaboration extended operational definitions

Construct (variable)	Operational definition levels	Explanations and examples
Fluency	1 = number of reinforced trials in a session	The number of trials in a session that receive a reward, i.e., the animal responded correctly to the cue
	2 = percent correct	The number correct out of the total number of trials in a session
	3 = total trials attempted in a session	The total number of trials in a session whether the responses were correct or incorrect (i.e., received an LRS if incorrect) from the animal to the trainer cue
	4 = trials completed before a repeat behavior	The number of correct trials offered by an animal before a repeat behavior, whether immediately repetitive or a second offering of a behavior given earlier in the session, is offered
Flexibility (energy)	1 = Low	Behavior performed with minimal effort (e.g., quiet whistle, small squirt)
	2 = Moderate	Behavior performed with moderate effort (e.g., tail slap with moderate water spray)
	3 = High	Behavior performed with effort (e.g., airplane, aggressive bark)
	4 = Homogeneous low	Multiple behaviors during trial that had the same low energy level (e.g., quiet whistle and small squirt performed together)
	5 = Homogeneous moderate	Multiple behaviors during the trial that had same moderate energy level (e.g., tail slap with moderate water spray and mid-volume whistle performed together)
	6 = Homogeneous high	Multiple behaviors during the trial that had the same high energy level (e.g., airplane and aggressive bark performed together)
	7 = Low + moderate	Separate behaviors of low and moderate energy level performed during the trial (e.g., small squirt and mid-volume bark performed together)
	8 = Low + high	Low and high energy level behaviors performed during the trial (e.g., quiet whistle and airplane performed together)
	9 = Moderate + high	Moderate and high energy level behaviors performed during the trial (e.g., mid-volume whistle and airplane performed together)
	10 = Low + moderate + high	Low, moderate, and high energy level behaviors performed during the trial (e.g., small squirt, mid-volume whistle, and aggressive bark performed together)
Flexibility (type)	1 = Motor	Behavior performed using body parts (e.g., pec slap, spy hop)
	2 = Vocal	Behavior performed involving noise (e.g., burp, whistle)
	3 = Bubble	Behavior performed using bubbles (e.g., bubbles, jacuzzi)
	4 = Homogeneous motor	Multiple motor behaviors performed during the trial (e.g., pec slap and spy hop performed together)
	5 = Homogeneous vocal	Multiple vocal behaviors performed during the trial (e.g., burp and whistle performed together)
	6 = Homogeneous bubble	Multiple bubble behaviors performed during the trial (e.g., bubbles and jacuzzi performed together)
	7 = Motor + vocal	Motor and vocal behaviors performed during the trial (e.g., spy hop and burp)
	8 = Motor + bubble	Motor and bubble behaviors performed during trial (e.g., pec slap and jacuzzi performed together)
	9 = Vocal + bubble	Vocal and bubble behaviors performed during the trial (e.g., burp and bubbles performed together)
	10 = Motor + vocal + bubble	Motor, vocal, and bubble behaviors performed during the trial (e.g., pec slap, burp, and jacuzzi performed together)
Flexibility (repertoire)	1 = in repertoire (yes)	A behavior that has been trained and is part of the animal’s known repertoire
	2 = not in repertoire (no)	A behavior that is not trained as part of the animal’s repertoire and not seen previously for this animal
Originality	1 = total actions produced only once across all sessions per animal	Number of simple actions and complex actions produced a single time by each animal across all sessions
	2 = total actions produced only once across all animals and all sessions	Number of simple actions and complex actions produced a single time by any animal across all sessions
	3 = action produced that was novel, not part of animal’s trained behavioral repertoire	Any action produced by an animal that was not part of its trained behavioral repertoire. i.e., a behavior that was not trained by a trainer to be under stimulus control
Elaboration	1 = single action	A single, simple behavior produced by the animal (e.g., spit, spy hop, vocal)
	2 = sequence of actions	Two or more behaviors produced in a series by the animal (e.g., vocal then headshake and pec wave) (Previously repeated single actions were acceptable in sequences.)
	3 = simultaneous behaviors	Two or more behaviors produced at the same time by the animal (e.g., vocal and head shake at the same time) (Previously repeated single actions were acceptable in sequences.)

Originality represents how unique an animal’s behavior is when compared with the repertoire of the individual, the group, population, or the species. As Guilford (1966) originally theorized, more common behaviors are less original, while less common actions are more original (Pryor et al., 1969). In our study, originality was evaluated by examining each performed behavior in comparison to previously exhibited behaviors of the individual (Table 2); the number of new-to-the-test session behaviors and the number of new-to-the-repertoire behaviors were documented during each test session for each killer whale.

In non-human animals, elaboration has not been formally evaluated and has only been speculated by Kaufman and Kaufman (2004). In the current study, we operationalized elaboration as a variation on a theme within a test session (e.g., offering different types of vocalizations: foghorn, scream, whistle, etc.) or offering complex or compound behaviors in response to one innovative S_D_ (e.g., ventral layback with water spit). To count as elaboration, these behaviors can be presented in sequence or simultaneously (Dudzinski et al. 2018). In this study, elaboration was measured as whether a single or multiple behaviors were performed at the same time (Table 2).

### Statistical analyses

For this study, because of the small sample, we did not emphasize individual animal differences but rather focused on all killer whales as a group to examine general trends in response to the innovate cue. To compare across all nine killer whales, only data from the first three test sessions were included in these analyses. Additionally, to allow for direct comparison across and between animals, data were standardized as follows: (1) frequency data were converted to proportions (i.e., frequency count per level were divided by a total number of reinforced trials); and (2) the least number of trials completed for any of the nine killer whales per session was used as the standard minimum number of trials to use for all killer whales (e.g., for session #1, TAK completed 9 trials, which was the minimum applied to all animals for session #1). All analyses used this standard minimum number of trials to compare all nine whales for statistical significance in potentially creative actions. Results when using all trials per killer whale per test session are included in the supplemental material. These analyses were repeated to cross validate the minimum standard sample with more robust sample size.

As an initial application of the constructs measured in creativity theory proposed by Guilford (1966) and standardized by Torrance (1974) in the development of the Torrance tests for creativity, we present data at the group level for the majority of the constructs (i.e., flexibility, originality, elaboration), but we present fluency at the individual level (session and animal) to demonstrate mastery of the task.

Binomial tests for the percent correct for fluency were assessed at the individual session and at the individual killer whale levels. Descriptive statistics were used to describe the remainder of the fluency results and originality results. Due to the individual variability visible in Table 4, originality results were reported descriptively only. To assess group effects, 4-way mixed ANOVA tests were conducted on calculated proportions with age class (immature, non-sexually mature and mature, sexually mature), sex (male and female), session (1–3), and a construct of interest operational definition (i.e., flexibility – 3 definitions, elaboration – 2 definitions). Huynh–Feldt corrections were reported because the assumption of sphericity was violated. Post hoc analyses were conducted if statistical significance was found for a model.

**Table 4:** Data from each animal for the minimum standard number of trials for each test session

Animal	Session #	Total # trials / session	Originality		Fluency				Flexibility																						Elaboration		
									Energy										Type										Repertoire				
			Total new across all sessions	Total Novel (not in rep.)	F1	F2	F3	F4	1	2	3	4	5	6	7	8	9	10	1	2	3	4	5	6	7	8	9	10	Yes	No	1	2	3
KAM	1	56			9	100%	9	9	1	3	1	1	0	0	3	0	0	0	1	4	0	1	0	0	3	0	0	0	8	1	5	4	0
	2	48	8, 28 +	0^	17	94.4%	18	11	4	2	2	3	1	0	2	3	0	0	3	2	2	2	0	0	5	3	0	0	17	0	7	8	2
	3	45			22	88.0%	25	19	3	1	4	4	0	0	3	6	0	1	3	2	3	3	0	1	1	8	1	0	22	0	8	11	3
KEI	1	141			9	100%	9	9	2	1	0	0	2	0	4	0	0	0	1	1	1	4	0	0	1	1	0	0	9	0	3	4	2
	2	47	9, 17	0	18	100%	18	18	1	11	0	2	0	0	3	0	1	0	7	4	1	3	1	0	2	0	0	0	18	0	12	5	1
	3	107			20	80.0%	25	5	4	10	3	1	1	0	0	1	0	0	8	8	1	0	0	0	2	1	0	0	18	2	17	1	2
SAK	1	12			7	77.8%	9	3	1	1	0	0	0	0	4	0	1	0	2	0	0	2	0	0	3	0	0	0	7	0	2	2	3
	2	54	8, 26	0	16	88.9%	18	5	1	4	0	3	0	0	4	3	0	1	5	0	0	7	0	0	2	1	0	1	16	0	5	8	3
	3	56			24	96.0%	25	22	7	5	2	1	0	1	5	1	1	1	9	4	1	5	1	0	4	0	0	0	24	0	14	8	2
MOA	1	107			9	100.0%	9	9	0	7	0	0	1	0	1	0	0	0	3	4	0	1	0	0	1	0	0	0	9	0	7	2	0
	2	123	18, 15	0	18	100.0%	18	18	1	6	1	0	5	2	1	0	2	0	7	1	0	6	1	0	2	0	1	0	18	0	8	7	3
	3	30			20	80.0%	25	4	2	14	0	0	0	1	1	1	1	0	12	3	1	3	0	0	1	0	0	0	19	1	16	4	0
TUA	1	22			8	88.9%	9	3	2	2	0	0	0	0	0	4	0	0	3	1	0	2	0	0	2	0	0	0	8	0	4	3	1
	2	18*	11, 14	0	12	66.7%	18	7	5	0	1	5	0	0	0	1	0	0	3	2	1	3	0	0	3	0	0	0	12	0	6	6	0
	3	34			22	88.0%	25	6	8	2	5	3	1	0	0	2	0	0	11	2	2	2	0	0	2	2	0	0	22	0	14	7	0
INO	1	67			7	77.8%	9	6	1	3	2	0	0	0	0	0	1	0	2	4	0	0	0	0	1	0	0	0	7	0	6	1	0
	2	77	5, 4	0	17	94.4%	18	3	0	8	4	0	3	0	1	0	1	0	3	8	1	0	0	0	1	1	3	0	17	0	12	3	2
	3	41			23	92.0%	25	19	7	16	0	0	0	0	0	0	0	0	2	16	5	0	0	0	0	0	0	0	23	0	23	0	0
KYU	1	23			9	100%	9	9	2	1	2	0	2	0	0	2	0	0	4	0	0	5	0	0	0	0	0	0	9	0	6	3	0
	2	24	13, 15	0	16	88.9%	18	7	5	0	4	1	0	2	0	4	0	0	9	0	0	7	0	0	0	0	0	0	16	0	9	6	1
	3	25*			23	92.0%	25	7	5	0	8	1	0	0	0	9	0	0	11	1	1	9	0	0	1	0	0	0	23	0	12	6	5
WIK	1	70			9	100%	9	9	2	2	1	1	2	0	1	0	0	0	2	3	0	3	0	0	1	0	0	0	9	0	5	4	0
	2	78	8, 17	0	13	72.2%	18	1	0	10	0	0	2	0	1	0	0	0	3	7	0	2	0	0	1	0	0	0	13	0	10	2	1
	3	99			22	88.0%	25	10	4	6	1	1	1	0	6	1	2	0	3	7	1	3	0	0	6	1	0	1	22	0	11	7	4
TAK	1	9*			8	88.9%	9	4	3	1	1	0	0	0	0	1	1	1	4	2	0	0	0	0	2	0	0	0	8	0	5	3	0
	2	38	9, 18	0	15	83.3%	18	12	3	3	0	1	0	0	1	3	3	1	4	2	0	5	0	0	3	1	0	0	14	1	5	7	3
	3	26			21	84.0%	25	14	7	7	1	8	0	0	3	1	0	0	7	2	0	7	0	0	4	1	0	0	21	0	9	9	3

Abbreviations are: F1 is the number of reinforced trials, F2 is fluency percent correct, F3 is total attempted trials, and F4 is the trials completed before a repeat behavior; flexibility energy and type columns 1 through 10 and elaboration 1, 2, and 3 are all defined in Table 3

^+^First number is single behaviors and second number after comma is complex behaviors (simultaneous or sequential actions, see Table 3 for definitions and examples)

^^^KAM produced a novel behavior but repeated it between session 1 and 2 and so it was removed from our count

*This is the minimum number of trials by any killer whale per session that identified the total number of trials to be used for each animal per session from which a direct comparison across all animals could be examined

## Results

Each killer whale responded distinctly during the creativity test sessions. Each killer whale produced a different number of trials per test session because of their individual responses to the innovative cue (i.e., some animals made three errors in a row sooner than other animals). Test session #1 had one animal produce 9 trials and all other animals produced more trials. Test session #2 had a different animal produce 18 trials and all other animals produced more trials. Test session #3 had yet another, different animal produce 25 trials while all other animals produced more trials. To address this variability, we used the minimum number of trials completed by one study animal during each of three test sessions to standardize data across all animals and allow for a direct comparison. The minimum number of trials for the three test sessions were 9 (test session #1), 18 (test session #2), and 25 (test session #3), respectively (Table 4).

**Fig. 1:**
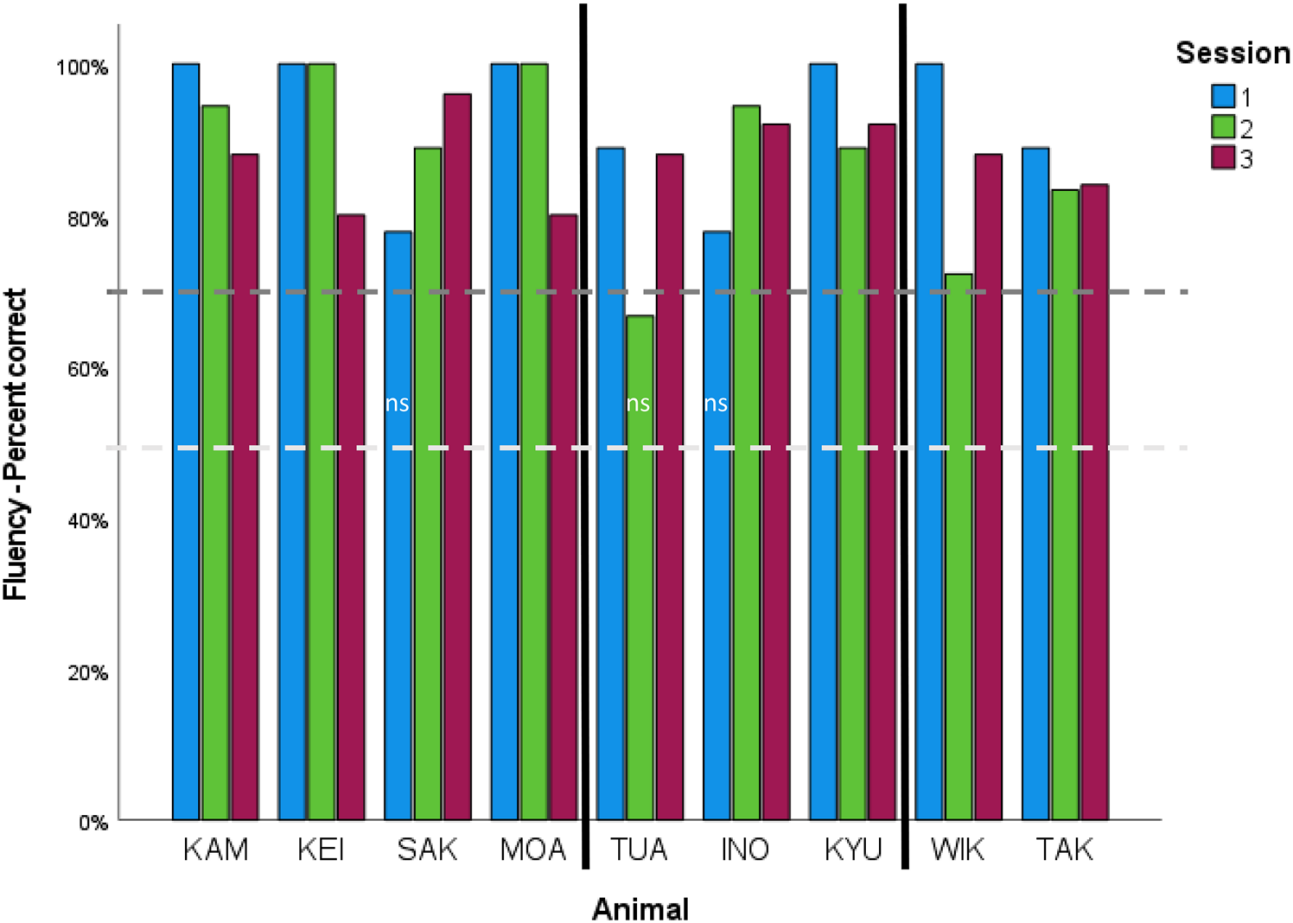
Fluency percent correct variable for the minimum standard number of trials (9, 18, 25 for sessions 1, 2, 3, respectively). *Note*: Binomial tests were performed: SAK & INO 7/9 (S1), *p* = .07; TUA 12/18, *p* = .07; WIK 13/18, *p* = .03; all values above 70% are significantly above chance, indicated with a dark gray line. The light gray dashed line represents the chance at 50%. Vertical black lines distinguish between animal groups based on social position: WIK and TAK are matriarchs in each study group; TUA, INO, KYU are the older males in the study; and KAM, KEI, SAK, and MOA are the immature animals

*Fluency (minimum number of trials) – Percent correct*: Binomial tests confirmed that all but three killer whales performed correctly above chance within sessions (Fig. 1, Table 4). In session 1, SAK and INO did not respond significantly above chance [*p* = 0.07, Fig. 1] while in session 2, TUA similarly did not respond significantly above chance [*p* = 0.07, Fig. 1]. All other animals, as well as these three individuals in other sessions, responded significantly above chance during testing (Fig. 1), demonstrating animal mastery of the task. We chose not to conduct group analyses here because all individuals performed well above chance (overall mean was 89%).

**Fig. 2:**
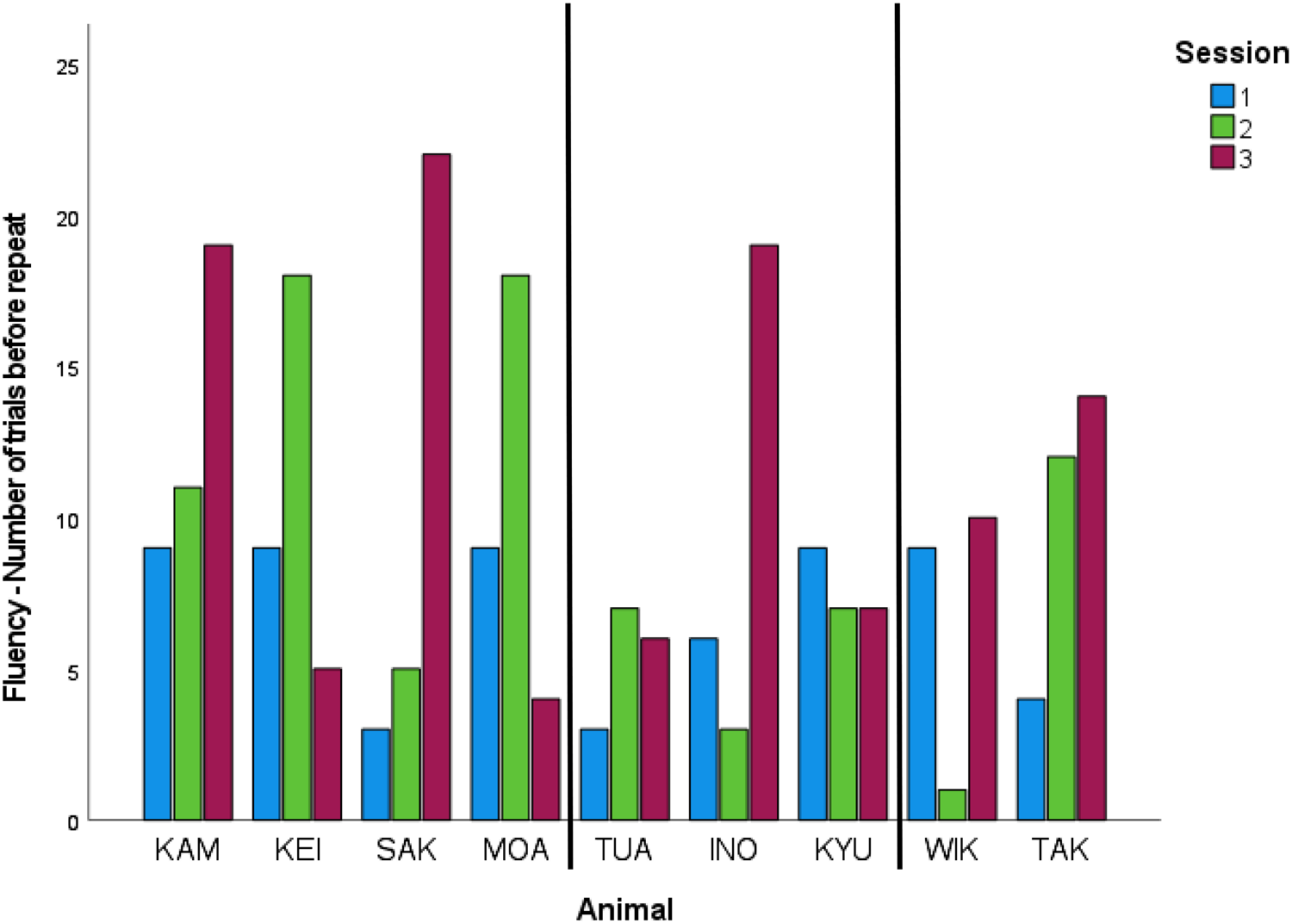
Fluency as the number of trials completed before a repeat behavior variable for the minimum standard number of trials (9, 18, 25 for sessions 1, 2, 3, respectively). Vertical lines indicate same details as in Fig. 1

*Fluency (minimum number of trials) – No. trials before repeat*: The trend was for most killer whales to show more consecutive correct trials before they repeated a behavior from session #1 to session #2 to session #3 (Fig. 2; Table 4). Departing from this trend were KEI and MOA, both immature males did not follow the overall trend of increasing the number of consecutive trials before a repeat behavior. Rather, KEI and MOA both showed early repeated behaviors in their third sessions (Fig. 2). Also, WIK and INO, both adults but of different sexes, showed an early repeat in session #2 but a dramatic increase in new behaviors before a repeat in session #3 (Fig. 2).

**Fig. 3:**
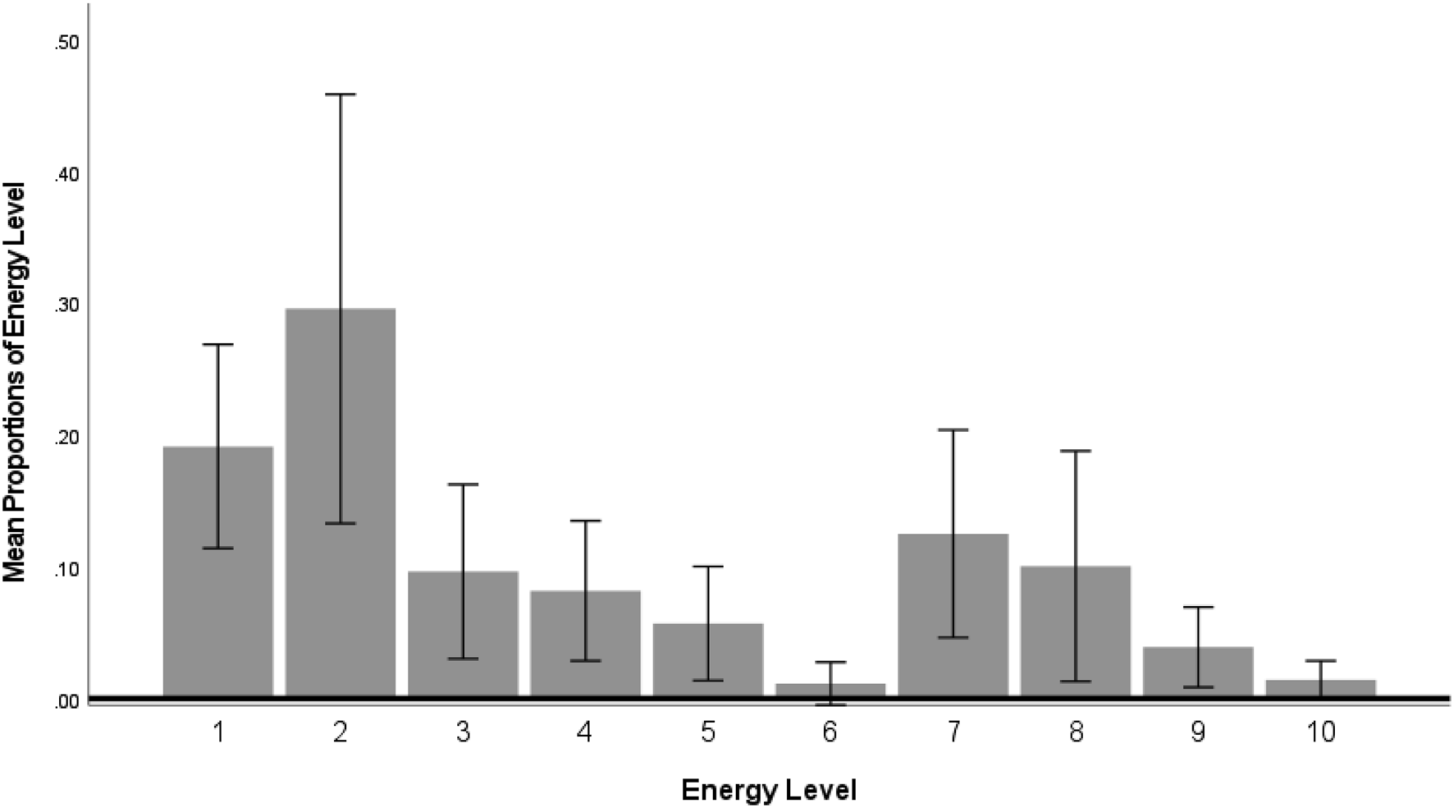
Flexibility—energy variable for the minimum standard number of trials (4-way mixed ANOVA: energy x session x sex x age classµ; sphericity not met). Energy level is defined in Table 3. Error bars represent 95% confidence intervals. Y-axis is mean proportion of energy level

*Flexibility-Energy*: Killer whales responded with low and moderate energy behaviors to the innovate cue significantly more than with high energy single actions, multiple behaviors of the same or mixed energy levels, as indicated by a significant main effect of energy [Huynh–Feldt *F*(4.26, 21.31) = 4.26, *p* < 0.001; partial eta sq = 0.629 (Fig. 3; Table 4)]. After low and moderate energy single action responses, the next most frequently used energy levels used were heterogenous—low and moderate then low and high—with multiple behaviors presented simultaneously or in sequence (Fig. 3). However, Sidak post hoc tests indicated that the killer whales performed significantly more low energy single actions than a combination of low and moderate energy action responses [*p* < 0.05]. No other significant differences were found.

**Fig. 4:**
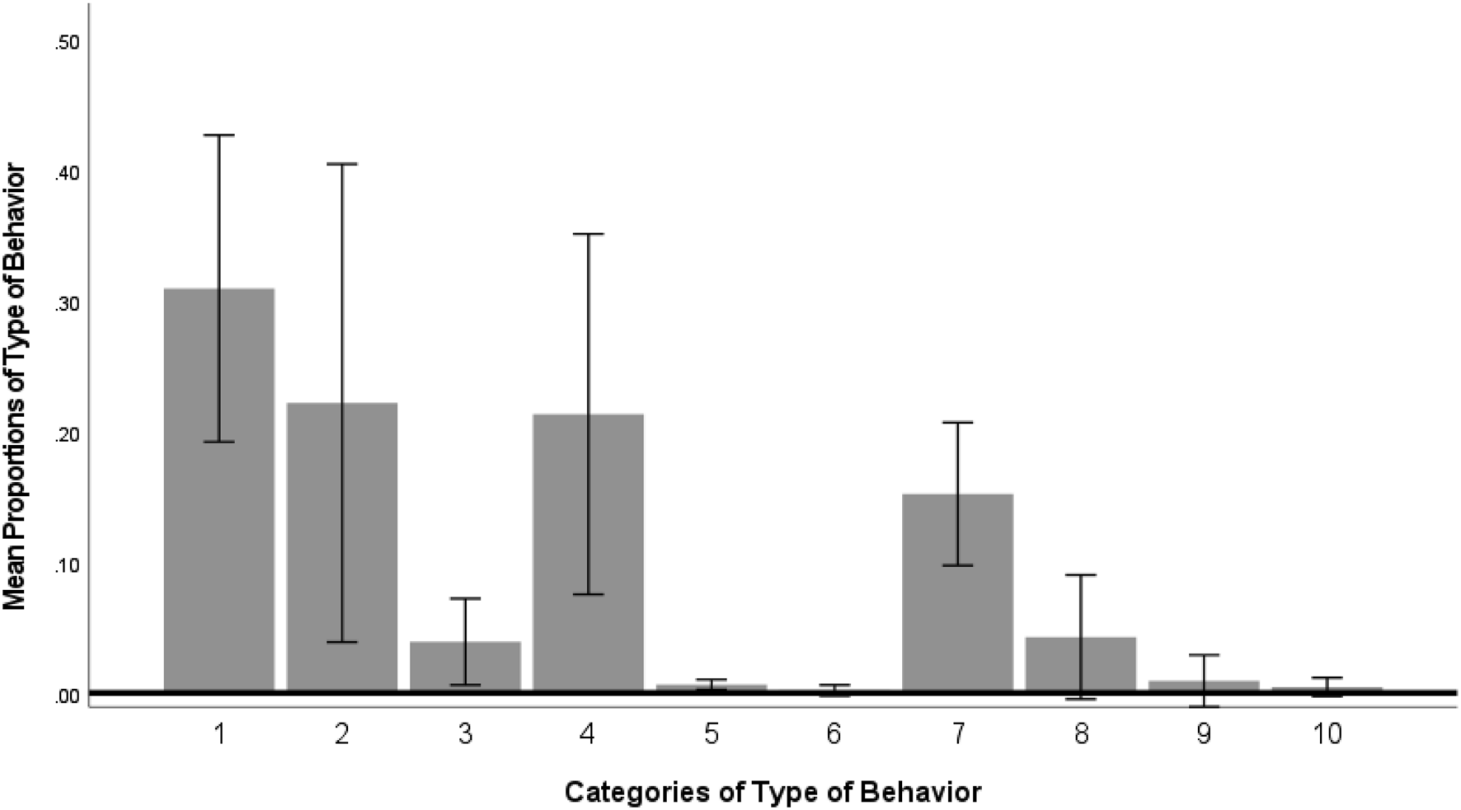
Flexibility – type variable for the minimum standard number of trials (4-way mixed ANOVA: energy x session x sex x age class). Type level is defined in Table 3. Error bars represent 95% confidence intervals. Y-axis is the mean proportion of the type of behavior

*Flexibility-Type*: Killer whales responded primarily with four types of flexibility: motor (type 1), vocal (2), multiple actions both/all motor (4), and motor and vocal actions together (7) as indicated by a significant main effect of type [Huynh–Feldt *F*(2.77, 13.85) = 10.58, *p* < 0.001; partial eta sq = 0.68 (Fig. 4; Table 4)]. Sidak post hoc analyses indicated that the killer whales were more likely to produce single motor behaviors than a combination of two or more vocal behaviors [*p* < 0.05] and a combination of all three types of behaviors [*p* < 0.05]. Sidak post hoc analyses also indicated that the killer whales performed significantly more combinations of motor and vocal actions than a combination of two or more vocal actions [*p* < 0.05], and a combination of two or more bubble actions [*p* < 0.05], and a combination of all three types of actions [*p* < 0.05]. Killer whales rarely used bubble responses or heterogenous behaviors that included bubbles and either motor or vocal actions (Fig. 4).

*Flexibility-Repertoire*: All animals responded with behaviors from their trained repertoire to most innovate cue requests (Table 4), though four killer whales (KAM, KEI, MOA, TAK) presented one to two behaviors each in a single session that had not previously been identified in their repertoires (Table 4). Three animals were immature – one female (KAM) and two males (KEI, MOA) – and the fourth individual was the matriarch from SWT (TAK) (Table 4). KAM displayed the non-repertoire action in session 1, while KEI and MOA both presented their non-repertoire behaviors in session 3, and TAK responded with a non-repertoire behavior in session 2.

*Originality*: From the minimum standard number of trials, all animals produced both single and complex actions (Table 5). Of the single behaviors, the number of actions performed a single time across each session ranged between animals from 5 to 18, with three males (MOA, KYU, TUA) producing the most single actions for all animals (Table 5). Complex behaviors, i.e., multiple single actions in series or simultaneously, were documented more for two young females (KAM, SAK; Table 5). When the minimum standard number of trials across the first three sessions for all animals were aggregated, each animal produced a range of actions that were unique to that individual (Originality 2, Table 5), with the two youngest females (KAM, SAK) producing the largest number of unique behaviors while one adult male (INO) produced the fewest unique actions (Originality 2, Table 5). Generally, all animals were similarly original in their production of different behaviors with the younger females and older males as outliers. Originality 2 merged both single and complex behaviors. For Originality 3, all actions exhibited by all animals were part of their behavioral repertoire (Table 5).

**Table 5:** Originality results for all nine killer whales for the minimum standard number of trials

Animal	Minimum standard number of trials			
	Originality 1		Originality 2	Originality 3
	Single	Complex		Un-trained
KAM	8	28	29	0
KEI	9	17	18	0
SAK	8	26	26	0
MOA	18	15	21	0
TUA	11	14	17	0
INO	5	4	7	0
KYU	13	15	16	0
WIK	8	17	20	0
TAK	9	18	19	0

In Originality 1, single and complex actions unless indicated in the untrained column (Originality 3) are part of each animal’s trained behavioral repertoire. Originality 2 is all animals all sessions and the number of actions each animal did

**Fig. 5:**
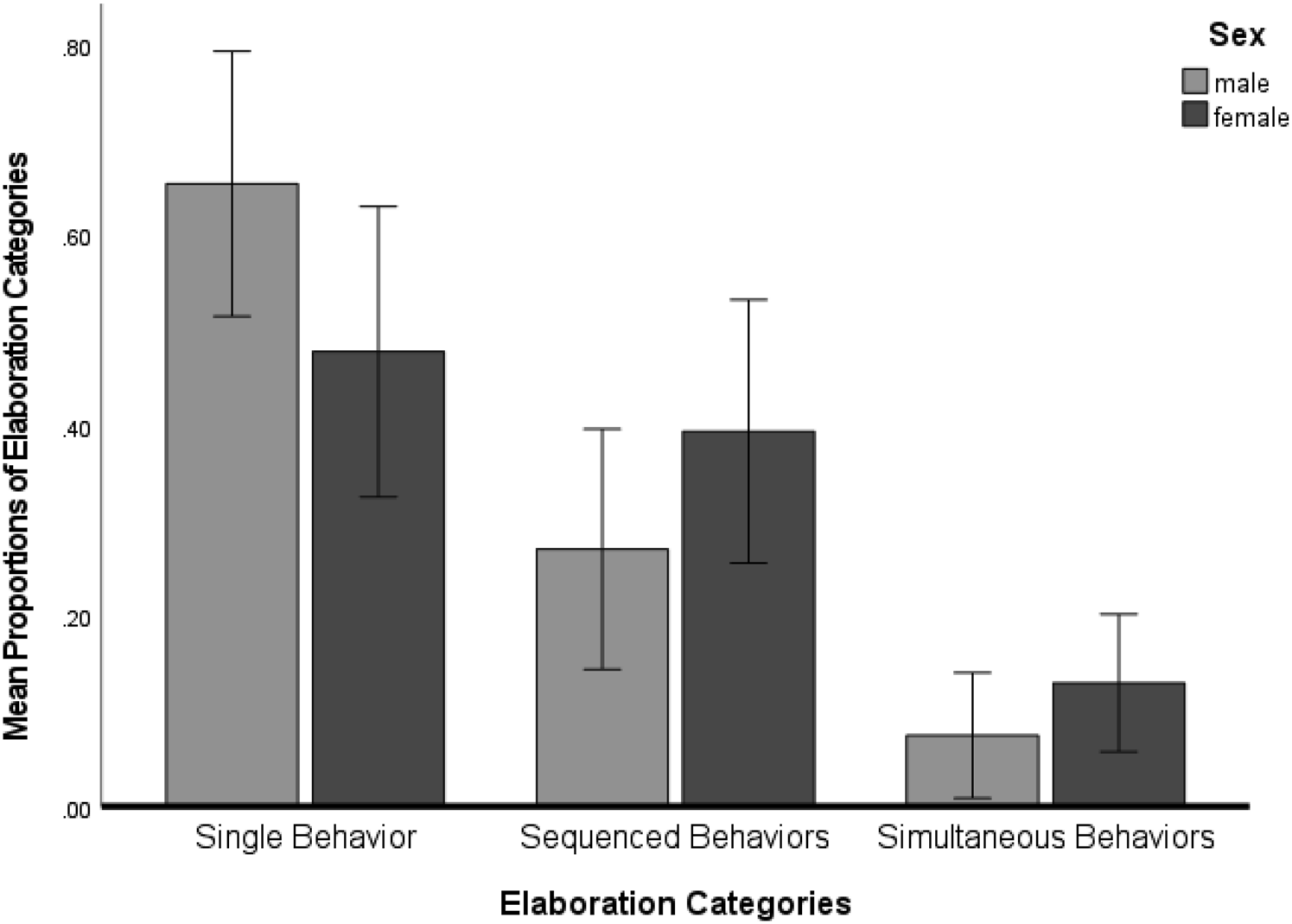
Elaboration of single, sequenced, or simultaneous behavior responses by killer whales according to sex for the minimum standard number of trials. Error bars represent 95% confidence intervals. Y-axis is the mean proportion of elaboration categories

*Elaboration*: All killer whales provided significantly more single behaviors than multiple actions (sequences or simultaneous) in response to the innovate cue, as indicated by a significant main effect for elaboration [Huynh–Feldt *F*(2, 10) = 32.58, *p* < 0.001; partial eta sq = 0.87]. Sidak post hoc tests indicated that the killer whales produced significantly fewer simultaneous behaviors (*M* = 0.10, SEM = 0.02) than either sequenced behaviors (*M* = 0.33, SEM = 0.04) [*p* < 0.01] or single action behaviors (*M* = 0.56, SEM = 0.04) [*p* < 0.001]. Single action behaviors and sequenced behaviors occurred with equal frequency. Both sexes responded similar to the overall trend of more single than multiple behaviors in response to the innovate cue; however, female killer whales seemed to produced more multiple actions than did males, although this result was not statistically significant at the 0.05 level [Huynh–Feldt *F*(2, 10) = 3.75, *p* = 0.06, partial eta sq = 0.43 (Fig. 5)].

### Cross validation of standardized trials

To cross-validate the results from the minimum standard number of trials (*N* = 9, 18, 25, respectively) during three test sessions per killer whale, we examined all trials per animal for each session (Table S1). These results are presented in the same order as above for the minimum standard number of trials (Fluency, Flexibility, Originality, and Elaboration).

*Fluency (all trials) – Percent correct*: A Binomial test confirmed that only TUA, in session 2, did not perform significantly above chance, though he did trend toward significance [*p* = 0.07; Fig. S1]. Using all trials in three sessions for all animals yielded responses at or above 68% as significantly above chance of 50% (Fig. S1).

*Fluency (all trials) – Number trials before repeat*: There was no overall trend in response between sessions for all killer whales with respect to when they exhibited their first repeat behavior (Fig. S2). Both matriarchs (TAK, WIK) had an increasing trend of more behaviors presented before a repeated action (Fig. S2). The older males (TUA, KYU, INO) presented different response patterns to each other and to the older females (Figure S2), with TUA and INO showing the most novel responses prior to a repeat in session 2 rather than either session 1 or 3. The younger killer whales seem to present a sex-specific response pattern; the young females (KAM, SAK) differed from each other though both showed more novel behaviors before a repeat in session 3 versus session 2 (Figure S2). The young males (KEI and MOA) exhibited a similar pattern that was opposite to the females: these males’ novel actions before a repeat behavior decreased from session 2 to session 3 (Fig. S2). These results support the existence of individual patterns of behavioral response across all nine killer whales to the innovative cue.

*Flexibility-Energy*: When all trials were included, killer whales responded with low and moderate energy behaviors to the innovate cue significantly more than with high energy single actions, multiple behaviors of the same or mixed energy levels, as indicated by a significant effect for energy [Huynh–Feldt *F*(3.75, 18.75) = 8.14, *p* < 0.001, partial eta sq = 0.62]. Sidak post hoc tests indicated that low energy single actions were performed significantly more often by the killer whales than two or more low energy behaviors [*p* < 0.01], two or more high energy behaviors [*p* = 0.052], two or more combinations of moderate and high energy behaviors [*p* < 0.05], and a combination of all three energy levels [*p* < 0.05]. Sidak post hoc tests also indicated that a combination of low and moderate energy actions was performed significantly more often than two or more high energy behaviors [*p* < 0.05], a combination of moderate and high energy behaviors [*p* < 0.05], and a combination of low, moderate, and high energy behaviors [*p* < 0.05]. Finally, one high energy behavior was produced more often than two or more high energy behaviors [*p* = 0.063], (Fig. S3﻿). After low and moderate energy single action responses, the next most frequently used energy levels used were heterogenous—low and moderate then low and high—with multiple behaviors presented simultaneously or in sequence (Fig. S3). These results for all trials are similar to those observed in the minimum standard trials (Fig. S3).

*Flexibility-Type*: Killer whales responded primarily with four types of flexibility: motor (type 1), vocal (2), multiple actions both/all motor (4), and motor and vocal actions together (7), which was indicated by a significant main effect of type [Huynh–Feldt *F*(2.77, 13.85) = 10.58, *p* < 0.001, partial eta sq = 0.68]. Sidak post hoc tests indicated that single motor actions were performed significantly more often than two or more vocal responses [*p* < 0.05] and all three types of actions were performed in combination [*p* < 0.05]. Additionally, the combination of motor and vocal actions were performed significantly more often than two or more vocal actions [*p* < 0.05], two or more bubble actions [*p* < 0.05], and a combination of motor, vocal, and bubble [*p* < 0.05 (Table 4; Fig. S4﻿)]. Killer whales rarely used bubble responses or heterogenous behaviors that included bubbles and either motor or vocal actions (Fig. S4﻿).

*Flexibility-Repertoire*: Similar to the standardized data, all animals responded with behaviors from their trained repertoire to the most innovative cue requests (Table ﻿S1), though four killer whales (KAM, KEI, MOA, TAK) presented one to two behaviors each in a single session that had not previously been identified in their repertoires (Table S1). Three animals were immature – one female (KAM) and two males (KEI, MOA) – and the fourth individual was the matriarch from SWT (TAK) (Table S1).

*Originality*: From all trials, all animals produced both single and complex actions (Table S2). Of the single behaviors, the number of actions ranged between animals from 9 to 38, with two males (MOA, KEI) and one dominant female (WIK) producing the most single actions for all animals (Table S2). The highest number of complex behaviors were demonstrated by the youngest animals both female (KAM, SAK) and male (MOA, KEI) and by one dominant female (WIK) (Table S2). When all trials across the first three sessions for all animals are aggregated, each animal produced a range of actions that were unique to that individual (All Trials Originality 2, Table S2), with the youngest animals (MOA, KAM, KEI, SAK) producing the largest number of unique behaviors while one dominant female (WIK) produced the fewest unique actions (Originality 2, Table S2). For Originality 3, the youngest female produced two behaviors not previously trained or part of her behavioral repertoire, while the other eight animals exhibited actions that were part of their behavioral repertoire (Table S2).

*Elaboration*: As found with the standardized data, when all trials for all killer whales were examined, significantly more single behaviors were presented than multiple actions (sequences or simultaneous) in response to the innovate cue [Huynh–Feldt *F*(2, 10) = 30.05, *p* < 0.001, partial eta sq = 0.6]. Sidak post hoc tests indicated that the animals performed significantly more single actions (*M* = 0.56, SEM = 0.04) than multiple actions performed simultaneously (*M* = 0.10, SEM = 0.01) [*p* < 0.01] or multiple actions performed sequentially (*M* = 0.33, SEM = 0.04) [*p* < 0.001]. However, there was no difference between single actions and sequenced actions. As seen in the standardized data, both sexes responded similarly to the overall trend of more single than multiple behaviors in response to the innovate cue. However, female killer whales produced more multiple (sequences and simultaneous) actions than did males [Huynh–Feldt *F*(2, 10) = 6.63, *p* = 0.02, partial eta sq = 0.57]. Sidak post hoc tests indicated that males produced significantly more single actions than females [*p* < 0.05] and females produced significantly more simultaneous actions than males [*p* < 0.01 (Fig. S5)].

## Discussion

Using four abstract constructs (fluency, flexibility, originality, elaboration), we confirmed that killer whales mastered the task, were flexible in their selection of behavior, performed a number of behaviors a single time, and were able to elaborate with sequences and simultaneous actions while under stimulus control, respectively. Much like humans, rough-toothed dolphins (Pryor et al. 1969; Pryor and Chase 2014), and bottlenose dolphins (Kuczaj and Eskelinen 2014b), killer whales can be creative in their behavior. When the results of this study are set within the larger context of previous literature (bottlenose dolphins: reinforcement history, Pryor and Chase 2014; training history and working memory, Lawrence et al. 2016; working memory, Mercado et al. 1998; long-term social memory, Bruck 2013; innovative behaviors, Kuczaj and Eskelinen 2014b, Pryor et al. 1969; killer whales: foraging strategies, Guinet 2011, Guinet and Bouvier 1995, Saulitis et al 2000, Similä and Ugarte 1993; short-term memory, Abramson et al. 2013, Abramson et al. 2018), the implication is that killer whales have the ability for cognitive processes that promote innovative responses to their environment and social lives.

To compare across all nine study animals, we limited our sample for statistical analyses to the minimum number of trials performed by an individual killer whale in each session. By doing so, we were able to directly compare performance on each of the constructs as they were examined from multiple operational definitions for all nine killer whales from two facilities. We recognized that the inclusion of all data would create confounds associated with the interpretation of the different constructs; however, we only used the full data for three sessions from each animal for validation of the limited sample.

We investigated multiple operational definitions to understand how best to measure these abstract constructs in non-human animals. Kaufman and Kaufman (2004) proposed the application of the TTCT to non-human animals, which was approached by Kuczaj and Eskelinen (2014b). Kaufman (2021) revisited the application of the TTCT constructs for non-human animals and marine mammals, in particular. In this essay on the theoretical presentation of the application of TTCT to non-human animals, Kaufman provides definitions that are derived directly from the operational definitions in the TTCT used with humans.

Fluency as the number trials completed was constrained for the first three sessions to the minimum number of trials to facilitate comparison across all study animals. Still, the number of trials increased across these three sessions for all animals. With the exception of two sessions for three different individuals, all animals performed significantly above chance for the percent correct response to the innovative S_D_, with 89% correct as of the overall average. This suggests that these killer whales understand the concept of “do something different/new,” which is one aspect of creativity. The only study for comparison is Kuczaj and Eskelinen (2014b) who used a different set of accepted behaviors for innovative. Still, our results align with those reported by Kuczaj and Eskelinen (2014b) for three bottlenose dolphins showing very consistent percent correct scores ranging from ~ 93.5% to ~ 94.5%.

As validation using all available trials, only one killer whale did not perform significantly above chance while all other animals performed at or above the overall average of 83%. When all data were included (see supplemental material), there was greater variability in overall fluency because there were a different number of total trials that each animal completed (see Supplemental Table 1). These results indicate that in general these killer whales were successful in following the rule of the innovate cue, which suggests that they understood the concept of innovative. Conversely, alternative explanations for performance on this task could suggest that killer whales successfully mastered response to the innovative cue (S_D_) without the need for a cognitive concept but through contingencies acquired with their experience(s). Future research should evaluate participant learning histories to determine if strategies based on contingencies could have been used to exhibit their knowledge. Variability was evident across the responses by individual killer whales and between ages and both sexes, which supports the notion that societal roles may require different levels of creativity or, at least, the ability to think flexibly. Killer whales reside in matriarchal societies with individuals likely assuming different roles depending on social activity (e.g., Esteban et al. 2015). The varied hunting strategies practiced by killer whales in different geographies (Kuczaj et al. 1998; Visser et al. 2007; Guinet 2011; Guinet and Bouvier 1995) likely requires flexibility not only in foraging behavior but in coordination with conspecifics. For example, adult female killer whales demonstrate the beaching foraging technique to their offspring before the latter successfully engage in this hunting strategy (Guinet 2011; Guinet and Bouvier 1995). The strand foraging technique has developed independently in at least two killer whale populations, which suggests that flexibility in foraging actions is present in this species. Bottlenose dolphins are also catholic foragers exhibiting flexibility in foraging behavior associated with different habitats [e.g., strand feeding in South Carolina (Duffy-Echevarria et al. 2008; Fox and Young 2012) and mud-plume feeding along the western Florida coast (Ramos et al. 2021)] and also in foraging with tools [e.g., sponging by dolphins in Monkey Mia, Australia (Smolker et al. 1997)].

With respect to fluency defined as the number of trials completed before a repeat behavior, there were some individual differences in patterns between sessions. For this creativity construct one would expect a linear increase in the number of novel responses to the innovative cue as support that the animal(s) understood the concept. A consistent linear response was not evident across all animals for both data samples. For example, two immature males decreased in the number of novel actions before repeating from the first two sessions to the third while one adult female and one adult male presented the fewest number of responses in the second session. These results highlight the aspect of variability across individuals that is present in tests of creativity; alternatively, these responses could simply indicate not enough sessions to observe a consistent trend. Other explanations such as the animals’ current level of motivation and social interactions cannot be ruled out.

Flexibility was operationalized in three different ways – energy level, type (motor, vocal, bubbles), and in the repertoire or not. There were no statistically significant interactions with age or sex on any of the operational definitions for flexibility, but there was a clear preference in actions performed for each flexibility variable. Individual differences were too variable to distinguish for all animals, so all animals were grouped for flexibility variable analyses. The moderate energy level with a single behavior was performed most frequently but with the most variability. This was followed by the low energy behaviors occurring significantly more often than other energy categories of single or complex (sequences or simultaneous actions) behaviors. The same pattern was evident when all data were included. Kuczaj and Eskelinen (2014b) found that two of their three dolphins preferred low energy behaviors with the third being more variable in energy level.

Preference in response for behavior type (see Table 3) was evident in all killer whales collectively, although there was no significant influence of age or sex on behavior type. Single motor actions and motor plus vocal actions were performed most followed by vocals and then two or more motor actions exhibited together. Kuczaj and Eskelinen (2014b) allowed for sequences of behaviors, which by definition created opportunities to perform multiple behavioral types within the same trial. Even so, our results align with more motor and vocal behaviors performed by all studied animals.

The killer whales performed behaviors primarily from their trained repertoire most frequently. However, four killer whales, three immature and one matriarch, each produced a novel or untrained, “creative” behavior during one of the first three sessions. These results align with those reported by Kuczaj and Eskelinen (2014b) who found that the individual dolphins produced “creative” behaviors. Given the difference in behavioral criteria between the research conducted with the three bottlenose dolphins and the killer whales, the bottlenose dolphins produced more creative behaviors than the killer whales with individual variability. We speculate that if the sequences of behaviors were evaluated for components in the performed behaviors, as Kuczaj and Eskelinen (2014b) did with the bottlenose dolphins then the killer whales would likely have similar results, and future research should examine this possibility.

Unlike the definitions used by Guilford (1966) and Torrance (1974) in their standardized creativity tasks in which responses are assessed qualitatively in terms of novel or rarity of a response, originality in the current study was defined as a relative frequency of the actions displayed by the animals. Kaufman (2021) and Kuczaj and Eskelinen (2014b) identified behaviors as creative if they were not in the trained behavioral repertoire but did not distinguish between novel actions that were produced once or produced and then repeated within or between sessions. In this study, two female killer whales each performed a single not-in-their-trained-repertoire action but then repeated that action in the same or a later session. Thus, while these two behaviors would be considered “novel” or “original”, these actions were removed from the originality analyses for each animal; however, the behaviors were retained in the flexibility-repertoire assessment.

Of all the behaviors exhibited in a session, individual animals varied in the number of unique actions performed only once in a session, with three males (2 adults, 1 juvenile) displaying the most single actions and two juvenile females producing the most complex behaviors. In bottlenose dolphins, young individuals play more, engage in varied forms of play, and increase the difficulty with which they play, especially when more same-aged conspecifics are available (Kuczaj et al. 2006). Although play has not specifically been studied in killer whales, an increase in play over the first three years of life was observed for a killer whale calf (Guarino et al. 2017), much like other odontocetes in which play has been studied (Greene et al. 2011; Hill and Ramirez 2014; Kuczaj et al. 2006). Additionally, from an evolutionary ecological viewpoint, young female killer whales should produce more complex behaviors given what is known about their eventual role in killer whale society as potential matriarchs (Bigg et al. 1990; Ford 2002).

Kaufman and Kaufman (2004) suggested that elaboration was a construct that could not be evaluated effectively for non-human animals using the standard human definition of developing ideas and adding details due to the constraints of the assessment procedure. To examine elaboration as a creativity construct in these non-human animals, we categorized the killer whale responses into single, sequential, and simultaneous behaviors. This approach was facilitated by the training protocol; during training, the animals were generally not allowed to put chains of behaviors together but were reinforced for performing a single or combination behavior. As expected, the killer whales performed single behaviors most frequently but did display sequential actions and simultaneous behavior combinations. Interestingly, this construct produced a sex difference with males performing more single actions than females and females performing more simultaneous behaviors than males when all data were included. For the minimum standard number of trials, this sex difference was trending but not significantly represented. In this study, elaboration as we defined it was likely impacted by the training protocol, as suggested by Kaufman and Kaufman (2004). That is, the current training protocol emphasized single action responses to be novel. Once animals understand the concept of “do something different/new,” then the training protocol could evolve to shift the criterion to emphasize compound or complex actions as the innovative requirement. Kuczaj and Eskelinen (2014b) did not distinguish between single actions and chained behaviors in their criterion; therefore, a direct comparison between their results and our findings in not currently possible.

### Implications

Individual differences have been confirmed in many animals (reviewed by Gosling 2001). Each animal will assume many roles in their society as they age; for example, in delphinids and whales, from dependent calf to precocious juvenile to autonomous adult (Bigg et al. 1990; Ford 2002; Highfill and Kuczaj 2010). The societal construct may also factor into the role an individual assumes; for example, killer whale adult females are matriarchs of their family pod (Bigg et al. 1990; Ford 2002). As such, they are responsible for knowledge transfer and group cohesion and movement (Beck et al. 2012; Ivkovich et al. 2010), and the matriarch’s role will be different to the males and younger females in her pod. These varied roles will be reflected in the different cognitive processes that require flexibility in thinking, which can be examined through studies into innovation like the one here.

Beck et al. (2012) put forth that sociality in killer whales is influenced by local ecological conditions, which indicates that adjusting to novel contexts in the environment or social structure is a valuable ability. Being able to readily accommodate to novel stimuli within one’s environment reflects an individual’s cognitive complexity and flexibility in thinking likely leads to increased survivability. These traits allow social species to not only survive a changing environment, both physical and social but also to thrive.

The need to thrive is also a consideration for killer whales in managed care. Training is enriching and can be cognitively challenging. Training an abstract concept, such as the innovative S_D_, elevates the mental challenge for both animal and animal trainer because effective communication of the abstract is required to balance success with failure, which simulates life in the wild, assuming expectations have been developed and are mutually understood by both parties. Translating this training process can elevate our care for these animals because it provides them with opportunities to make choices. They decide on the type, energy, and complexity of behavior with which they respond. This choice returns control of a situation to the animal, which contributes to their positive welfare.

### Limitations and future directions

Dudzinski et al. (2018) suggested that behaviors produced in training sessions could be influenced by individual differences in the trainers and animals, schedules of reinforcement, and differential reinforcement (i.e., magnitude and preference). Individual killer whales may respond differently to different trainers, which can cause inconsistencies in performed behaviors. A killer whale’s motivation can also impact the behaviors they produce. Lack of motivation could result in lower energy behaviors, therefore a session with low motivation would be reflected in the test session data (Dudzinski et al. 2018).

Each animal had different experiences in terms of the number of trials that were allowed to be completed with one facility offering more opportunities than the other. Although this difference may also have been driven by the individual killer whale; it should be standardized across testing sessions to promote consistency in response and interpretation. The definition of percent correct is one way this standardization can be addressed, but it is still subject to the number of trials made available to the animal.

To facilitate comparison across all nine animals, we used a limited set of data. While limiting the data did not affect the results significantly, some variation across the constructs between the minimum standard number of trials and all trials for the first three sessions did occur. For example, fluency-percent correct was lower when all data were included, suggesting reduced variability with the limited sample. Also, no significant interactions were observed for any of the analyses except for elaboration, and only with all trials. Additional trials per session and additional sessions per animal could offer more consistent results.

We modified the definitions of the TTCT abstract constructs to allow for an examination of creativity in non-human animals using the four constructs with animals under stimulus control. As such, our results are not directly comparable to the literature on human creativity. This initial investigation into the construct of creativity from an androcentric perspective provides many opportunities for future examination of non-human innovation. Areas to expand upon include refinement of definitions, testing under timed conditions such as those conducted with humans, and assessing the effects of additional training and increased expectations of behavioral responses. If significant responses to these constructs are evident, suggesting non-human animals understand the concept of innovation, then a future examination of compound behaviors—whether sequences or simultaneous actions—may offer insight into the cognitive processes underlying creativity in non-human animals.

Animals have myriad ways of expressing creativity, but we must expand our human definition of creativity so that we do not only use human expressions of creativity to define and understand non-human animals. We should emphasize the animal’s perspective, not anthropocentric views.

## Supplementary Information

Below is the link to the electronic supplementary material.

Supplementary file1 (DOCX 102 KB)

Supplementary file2 (PDF 134 KB)
