# Supplementary material for: Killer whale innovation: teaching animals to use their creativity upon request
Source: Anim Cogn. 2022 Sep 20;25(5):1091–108. doi: 10.1007/s10071-022-01635-3 (PMC9617837; doi:10.1007/s10071-022-01635-3)
Supplement: Supplementary file 1 — Supplementary file1 (DOCX 102 KB) [file 10071_2022_1635_MOESM1_ESM.docx]

**Supplemental Figures 1-5 and Supplemental Table 2**

ns


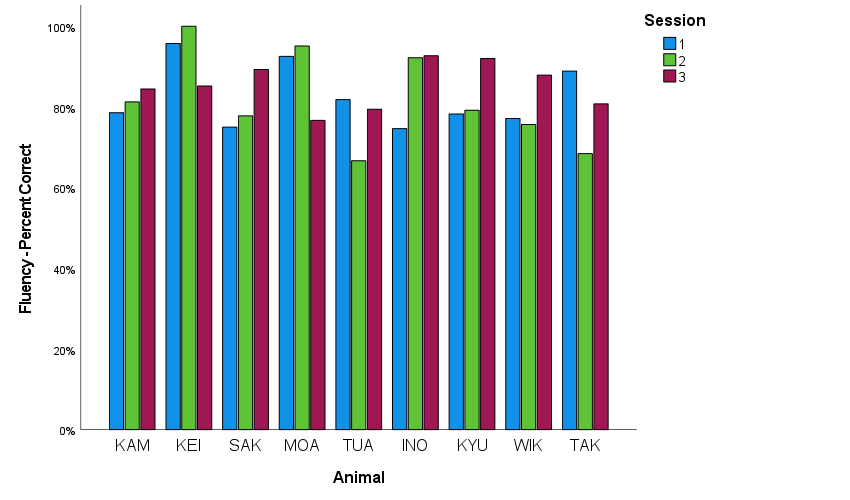


**Figure S1.** Fluency percent correct variable for all trials for all three test sessions for all animals. *Note*: Binomial tests: TUA 12/18, p = .07; TAK 26/38, p < .01; all values above 68% are significantly above chance. NS indicates not significantly above chance. Dashed white line denotes 50% chance level. Vertical black lines distinguish between animal groups based on social position in both graphs: WIK and TAK are matriarchs in each study group; TUA, INO, KYU are the older males in the study; and KAM, KEI, SAK, and MOA are the immature animals.


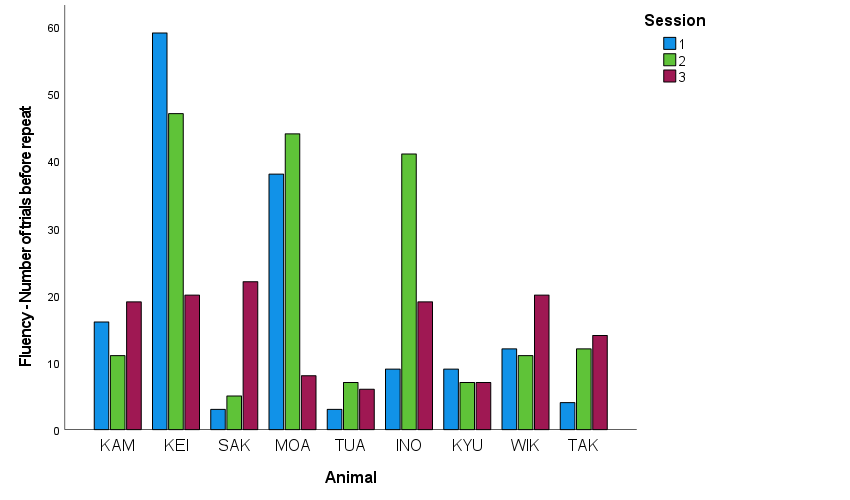


ns

**Figure S2.** Fluency as the number of trials completed before a repeat behavior variable for all trials for all three test sessions for all animals. Vertical lines indicate same details as in Figure 1.


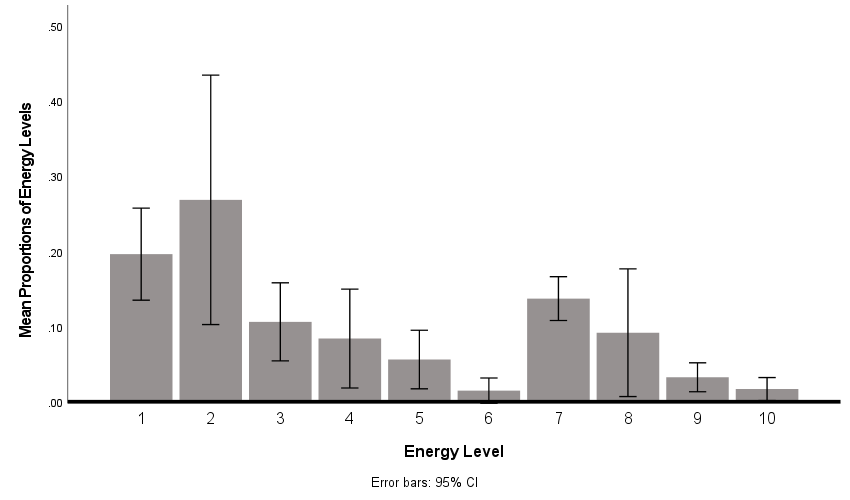


**Figure S3.** Flexibility - energy variable for all trials (4-way mixed ANOVA: energy x session x sex x age class). Energy level is defined in Table 3.


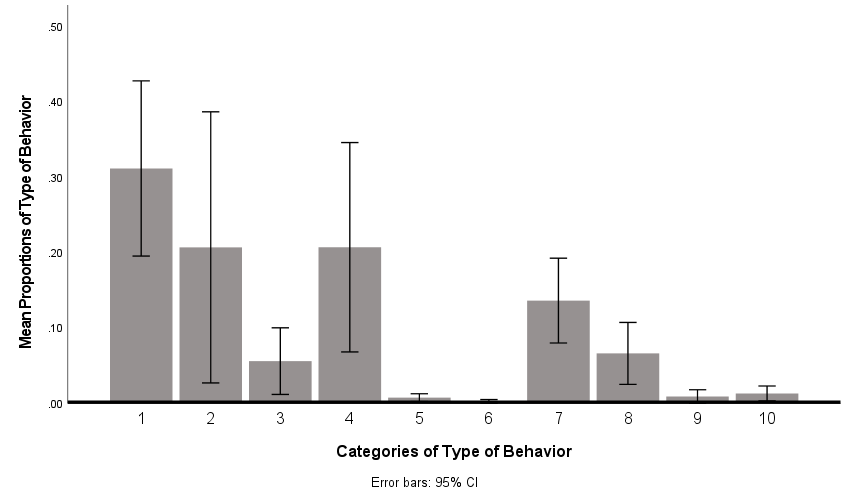


**Figure S4.** Flexibility – type variable for all trials (4-way mixed ANOVA: energy x session x sex x age class). Type level is defined in Table 3.


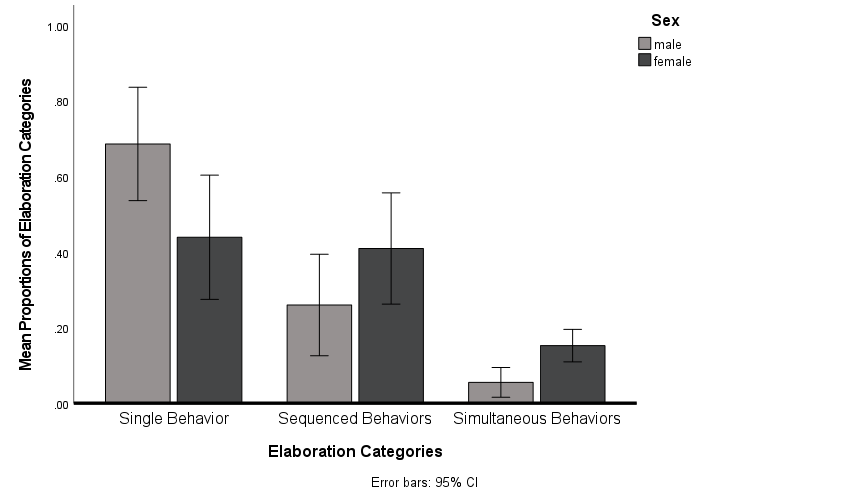


**Figure S5.** Elaboration of single, sequenced, or simultaneous behavior responses by killer whales according to sex for all trials.

**Table S2.** Originality results for all nine killer whales for all trials. In Originality 1, single and complex actions unless indicated in the untrained column (Originality 3) are part of each animal’s trained behavioral repertoire. Originality 2 is all animals all sessions and the number of actions each animal did. See Table 3 for definitions of each originality construct.

|  | **All Trials** | | | | |
| --- | --- | --- | --- | --- | --- |
|  | Originality 1 | | Originality 2 | Originality 3 |  |
| Animal | Single | Complex |  | Un-Trained |  |
| KAM | 17 | 74 | 74 | 2 |  |
| KEI | 35 | 56 | 66 | 0 |  |
| SAK | 13 | 62 | 62 | 0 |  |
| MOA | 38 | 64 | 79 | 0 |  |
| TUA | 14 | 18 | 21 | 0 |  |
| INO | 23 | 23 | 30 | 0 |  |
| KYU | 13 | 18 | 18 | 0 |  |
| WIK | 25 | 60 | 5 | 0 |  |
| TAK | 9 | 27 | 27 | 0 |  |
