# Supplementary material for: Killer whale innovation: teaching animals to use their creativity upon request
Source: Anim Cogn. 2022 Sep 20;25(5):1091–108. doi: 10.1007/s10071-022-01635-3 (PMC9617837; doi:10.1007/s10071-022-01635-3)
Supplement: Supplementary file 2 — Supplementary file2 (PDF 134 KB) [file 10071_2022_1635_MOESM2_ESM.pdf]

Table S1. Data from each animal for all trials for each test session. Abbreviations are: F1 is the number of reinforced trials, F2 is fluency percent correct, F3 is total attempted trials, and F4 is the trials completed before a repeat behavior; flexibility energy and type columns 1 through 10 and elaboration 1, 2, and 3 are all defined in Table 3.

			Originality		Fluency				Flexibility																						Elaboration		
Animal	Session #	Total # trials / session	Total new across all sessions	Total Novel (not in rep.)	F1	F2	F3	F4	Energy										Type										Repertoire				
									1	2	3	4	5	6	7	8	9	10	1	2	3	4	5	6	7	8	9	10	yes	no	1	2	3
	1	56			44	78.6%	56	16	7	8	1	7	3	0	13	1	1	3	9	7	0	6	0	0	14	5	1	2	42	2	15	22	7
KAM	2	48	6, 68+	1^	39	81.3%	48	11	8	6	4	4	1	1	7	6	1	1	7	5	5	2	0	0	10	10	0	0	38	1	16	13	10
	3	45			38	84.4%	45	19	4	2	6	6	3	0	9	7	0	1	4	3	3	9	0	1	5	11	2	0	38	0	11	20	7
	1	141			135	95.7%	141	59	26	51	6	7	14	0	27	1	2	1	32	37	15	28	1	0	7	12	2	1	134	1	84	39	12
KEI	2	47	14, 52	0	47	100%	47	47	7	28	2	3	1	0	5	0	1	0	12	21	4	5	1	0	3	1	0	0	47	0	37	8	2
	3	107			92	85.2%	108	20	9	48	19	2	6	3	1	1	3	0	27	36	13	4	1	0	3	8	0	0	88	4	76	12	4
	1	12			9	75.0%	12	3	1	1	0	0	1	0	5	0	1	0	2	0	0	4	0	0	3	0	0	0	9	0	2	4	3
SAK	2	54	3, 59	0	42	77.8%	54	5	3	4	5	5	1	1	10	11	0	2	11	1	0	17	0	0	6	3	0	4	42	0	12	26	4
	3	56			50	89.3%	56	22	11	7	6	2	0	2	7	10	3	2	14	7	1	12	1	0	4	10	0	1	49	1	22	16	12
	1	107			99	92.5%	107	38	11	40	15	0	13	2	13	1	3	1	38	24	4	15	2	0	14	1	1	0	100	0	66	21	12
MOA	2	123	22, 57	1	117	95.1%	123	44	11	41	19	3	14	4	15	2	7	1	54	16	2	26	1	0	12	3	3	0	117	0	71	38	8
	3	30			23	76.7%	30	8	3	14	2	0	0	1	1	1	1	0	14	4	1	3	0	0	1	0	0	0	22	1	19	4	0
	1	22			18	81.8%	22	3	6	3	0	1	0	0	3	5	0	0	5	2	2	3	0	0	4	1	0	1	18	0	9	8	1
TUA	2	18	7, 14	0	12	66.7%	18	7	5	0	1	5	0	0	0	1	0	0	3	2	1	3	0	0	3	0	0	0	12	0	6	6	0
	3	34			27	79.4%	34	6	9	4	5	4	1	0	2	2	0	0	13	2	3	5	0	0	2	2	0	0	27	0	17	10	0
	1	67			50	74.6%	67	9	9	21	10	4	2	1	1	1	1	0	8	28	4	2	0	0	5	2	0	1	48	2	41	8	1
INO	2	77	11, 19	0	71	92.2%	77	41	11	35	13	1	6	1	3	0	1	0	12	39	8	4	1	0	2	2	3	0	71	0	59	9	3
	3	41			38	92.7%	41	19	13	23	0	1	0	0	1	0	0	0	6	24	6	0	0	0	0	2	0	0	37	1	36	2	0
	1	23			18	78.3%	23	9	4	2	4	0	2	0	3	3	0	0	9	0	0	9	0	0	0	0	0	0	18	0	11	6	1
KYU	2	24	6, 12	0	19	79.2%	24	7	5	0	4	1	0	3	0	6	0	0	9	0	0	10	0	0	0	0	0	0	19	0	9	9	1
	3	25			23	92.0%	25	7	5	0	8	1	0	0	0	9	0	0	11	1	1	9	0	0	1	0	0	0	23	0	12	6	5
	1	70			54	77.1%	70	12	9	19	3	4	10	0	6	1	2	0	12	17	3	10	2	0	5	3	1	2	54	0	31	20	3
WIK	2	78	8, 51	0	59	75.6%	78	11	9	32	1	4	7	0	5	0	1	0	21	18	3	7	0	0	8	2	0	0	59	0	42	15	2
	3	99			87	87.9%	99	20	13	31	4	6	9	0	20	1	3	0	19	23	6	14	1	0	17	4	1	2	86	1	48	24	15
	1	9			8	88.9%	9	4	3	1	1	0	0	0	0	1	1	1	4	2	0	0	0	0	2	0	0	0	8	0	5	3	0
TAK	2	38	3, 24	0	26	68.4%	38	12	6	3	2	3	0	0	3	5	3	1	9	2	0	9	0	0	5	1	0	0	25	1	10	12	4
	3	26			21	80.8%	26	14	7	1	1	8	0	0	3	1	0	0	7	2	0	7	0	0	4	1	0	0	21	0	9	9	3

+ first number is single behaviors and second number after comma is complex behaviors (simultaneous or sequential actions, see Table 3 for definitions and examples).

^KAM produced a novel behavior but repeated it between session 1 and 2 and so it was removed from our count.
